# Explainable machine learning can outperform Cox regression predictions and provide insights in breast cancer survival

**DOI:** 10.1038/s41598-021-86327-7

**Published:** 2021-03-26

**Authors:** Arturo Moncada-Torres, Marissa C. van Maaren, Mathijs P. Hendriks, Sabine Siesling, Gijs Geleijnse

**Affiliations:** 1 Department of Research and Development, Netherlands Comprehensive Cancer Organization (IKNL), Zernikestraat 29, 5612 HZ Eindhoven, The Netherlands; 2 Department of Health Technology and Services Research, University of Twente, Enschede, The Netherlands; 3 Department of Medical Oncology, Northwest Clinics, Alkmaar, The Netherlands

**Keywords:** Breast cancer, Scientific data, Computer science

## Abstract

Cox Proportional Hazards (CPH) analysis is the standard for survival analysis in oncology. Recently, several machine learning (ML) techniques have been adapted for this task. Although they have shown to yield results at least as good as classical methods, they are often disregarded because of their lack of transparency and little to no explainability, which are key for their adoption in clinical settings. In this paper, we used data from the Netherlands Cancer Registry of 36,658 non-metastatic breast cancer patients to compare the performance of CPH with ML techniques (Random Survival Forests, Survival Support Vector Machines, and Extreme Gradient Boosting [XGB]) in predicting survival using the $c$-index. We demonstrated that in our dataset, ML-based models can perform at least as good as the classical CPH regression ($c$-index $\sim \,0.63$), and in the case of XGB even better ($c$-index $\sim 0.73$). Furthermore, we used Shapley Additive Explanation (SHAP) values to explain the models’ predictions. We concluded that the difference in performance can be attributed to XGB’s ability to model nonlinearities and complex interactions. We also investigated the impact of specific features on the models’ predictions as well as their corresponding insights. Lastly, we showed that explainable ML can generate explicit knowledge of how models make their predictions, which is crucial in increasing the trust and adoption of innovative ML techniques in oncology and healthcare overall.

## Introduction

The Cox Proportional Hazards (CPH) model^1^ is the most frequently used approach for survival analysis in a wide variety of fields^2^. In oncology, it is mainly used to identify the prognostic factors that have an impact on patients’ recurrence or survival^3,4^.

Although the CPH model has been widely adopted by the scientific community^2,4^ due to its ease of use, fast computation, and—most importantly—meaningful output, it inherently presents a few shortcomings. For instance, it is an inadequate model for high dimensional settings (i.e., when the number of features exceeds the number of data instances). Moreover, CPH regression relies on a few other restrictive assumptions, such as proportionality of the hazard functions for any two patients (i.e., their ratio is constant over time) and uncorrelated features. Last but foremost, it is unable to properly model nonlinearities and interaction effects (which are often present in data) out-of-the-box.

In the last years, machine learning (ML) has proven to be a great complement to traditional statistical methods for improving cancer diagnosis, detection, prediction, and prognosis^5–10^. To alleviate the aforementioned problems of CPH-based modelling, several ML techniques capable of accounting for interaction effects and nonlinearities have been successfully adapted to handle censored data, spreading its use for survival analysis of a wide variety of tumors^2^. Actually, several studies have consistently found that ML-based approaches are capable of performing at least as good as conventional CPH analysis in predicting patient survival^11–17^. Unfortunately, these studies often treat ML models as black-boxes. This is undesirable, since it makes it difficult to understand how a model obtained its predictions. More importantly, this limits the trust that patients and clinicians have on the models’ predictions^18^.

ML explainability techniques aim to provide an explanation of *how* a ML model reached its output in understandable terms^19^. Moreover, they can help verify certain traits that are important to a (ML) model, such as robustness, fairness, and agreement with human (clinical) intuition^20^. ML explainability is especially important in healthcare: if decisions need to be made that are (at least partially) based on predictions made by ML algorithms, users need to be able to understand how the algorithm came up with that decision to trust and, more importantly, adopt the model.

In this paper, we compared the performance of the classical CPH model with several ML techniques in predicting breast cancer survival. We evaluated the models’ performance using Harrell’s concordance index. More importantly, we used Shapley Additive exPlanation (SHAP) values to shine some light on the performance of the classical CPH regression and of the best-performing ML technique, facilitating their interpretation.

## Methods

### Data

Data were obtained following the standard data usage request process from the Netherlands Cancer Registry (NCR). The NCR is a nationwide population-based registry including all newly diagnosed malignancies from 1989 on. Patient-, tumor-, and treatment-related characteristics are directly registered from patient files by trained data managers. Since this study had a national, non-interventional retrospective design and all data were analyzed anonymously, individual informed consent was waived by the NCR Supervisory Committee. After ethical approval of the proposed methods also by the NCR Supervisory Committee, the data were granted under request K18.999. All methods in this study were carried out in accordance with relevant guidelines and regulations.

We selected all female patients in the Netherlands diagnosed between 2005 and 2008 with primary invasive non-metastatic breast cancer who underwent curative surgery (breast-conserving surgery or mastectomy). Features included age, tumor characteristics, (hormonal) receptor statuses, clinical and pathological TNM-staging (as defined by the 6th edition of the Union for International Cancer Control TNM Classification), and number of removed and positive lymph nodes.

### Pre-processing

We used the same steps of pre-processing and cleaning the data used in our previous work^21^. First, we performed imputation of the input features using a multivariate single approach, since it proved to be a sound method with a good performance and acceptable computational time (the latter which is especially important when dealing with a large number of patient records, as in the dataset at hand). We used the Python package DataWig (v. 0.10)^22^ to train a neural network to predict the missing values of each feature using information from other features that were at least correlated moderately (i.e., absolute correlation value $\ge 0.5$)^23^. These networks were parameterized with a number of nodes of 100, batch size of 16, epoch number of 100, patience of 5, and use *relu* as an activation function. In the case of continuous variables, they had one hidden layer and used a squared loss function. In the categorical case, they had as many hidden layers as labels of the imputed variable and used a cross-entropy loss function. All networks used Adam as an optimizer with a $\eta$ of 0.004, $\beta _1$ of 0.9, $\beta _2$ of 0.999, and $\epsilon$ of $1\times 10^{-8}$. In the instances where this approach failed (e.g., if there were not enough correlated features to train the neural network), we used *k*-Nearest Neighbor. In the case of continuous/categorical variables, we used an euclidean/jaccard distance metric and looked at the $k=10$ nearest neighbors of a data point. If at least three of them were not missing values, we performed the imputation using their mean/mode^24,25^. Otherwise, the overall mean/mode was used. In all cases, the imputation was performed using exclusively the original data available. In other words, we did not use imputed data to impute other features. The output variables (i.e., censorship status and time to event) were complete and required no imputation whatsoever.

Next, we engineered three new features that make sense for clinical practice and thus we thought would be relevant for the task at hand: the ratio between positive and removed lymph nodes (continuous, with a range between 0 and 1), a summary of ER, PR, and HER2 statuses (i.e., triple positive [ER+, PR+, HER2+], hormone-receptor positive [ER+ and/or PR+], hormone-receptor negative [ER−, PR−, HER2+], triple-negative [ER−, PR−, HER2−]; categorical), and an indicator of whether all receptor statuses were negative or not (i.e., triple negative; categorical dichotomous). Since these features were engineered from the complete data, they presented no missing values.

Lastly, we performed feature selection by combining the results of two different approaches: one based on algorithms and one based on clinical expertise. On the one hand, for the algorithm-based approach, we used a combination of 21 feature selection methods. Each of them yielded a ranking of feature predictiveness. Then, we calculated the median and chose the six best ranked features, since in our previous work^21^ we found that for this particular dataset, going above that number did not improve the model’s accuracy. On the other hand, we defined an additional set of six features (in no particular order) that were deemed to be the most predictive according to clinical experts. The union of both sets resulted on the final dataset, consisting of 9 features of 36,658 patients.

Table 1 shows a summary of the data. For continuous variables, we report their mean and standard deviation; for categorical variables, we report their absolute and relative numbers (as a percentage). Moreover, we also show the completeness of the original features (i.e., before imputation). Lastly, we also show which features were obtained from the algorithm-based approach (including their ranking) and which were included by suggestion of clinical experts. These data are not publicly available due to privacy restrictions, but are available on reasonable request.

**Table 1:** General overview of the data used for this study.

Feature	Mean	(SD)	N	(%)	Completeness	Feature selection	
					[%]	Algorithm	Clinical
***age***							
Age at diagnosis (years)	60.25	(13.96)			100	✓(1)	✓
ratly*							
Ratio between positive and removed lymph nodes	0.11	(0.22)			100	✓(2)	
***rly***							
No. of removed lymph nodes	8.13	(7.84)			98.7	✓(3)	
***ptmm***							
Tumor size [mm]	20.32	(13.79)			87.1	✓(4)	✓
***pts***							
Pathological tumor stage					100	✓(5)	
I			15,412	(42.04)			
IIA			10,848	(29.59)			
IIB			4766	(13.00)			
IIIA			3145	(8.57)			
IIIB			782	(2.13)			
IIIC			1705	(4.65)			
***grd***							
Tumor grade					89.8	✓(6)	✓
1 (well differentiated)			7563	(20.63)			
2 (moderately differentiated)			17,926	(48.89)			
3 (poorly differentiated)			11,169	(30.46)			
***mor***							
Tumor morphology					100		✓
Ductal			29,473	(80.39)			
Lobular			4109	(11.20)			
Mixed			1464	(3.99)			
Other			1612	(4.40)			
***ply***							
No. of positive lymph nodes	1.53	(3.53)			97.6		✓
rec*							
Receptor status					100		✓
Triple+			1983	(5.40)			
HR+			28,170	(76.84)			
HR−			2048	(5.58)			
Triple−			4457	(12.16)			

Features marked with an asterisk were engineered (see text for more details). The number in parenthesis in the *Algorithm* column corresponds to the feature’s ranking given by the feature selection. *SD* standard deviation, *HR +* hormone receptor positive, *HR -* hormone receptor negative.

### Models

#### Cox proportional hazards

We performed a conventional multiple CPH regression. The CPH model is a semiparametric approach that computes the impact of a set of given covariates (i.e., features) on the hazard (i.e., risk) of an event occurring (in our case, death)^26^. In this case, the hazard of a patient is a linear function of a population baseline hazard (that changes over time) and of his/her static predictor covariates (multiplied by their corresponding coefficients). It makes no assumptions of the underlying hazard function.

We also used the CPH model to predict patient ranking using risk scores.

#### Machine learning models

There is a growing number of ML models for survival analysis^2,27^. Similarly to the CPH case, we predicted patient survival ranking using three of them: Random Survival Forests (due to their frequent use in literature relevant to this study^11,13–15^), Survival Support Vector Machines (given that their use of kernels to map survival in high-dimensional spaces makes them an attractive option^16,28^), and Extreme Gradient Boosting (since in addition to its execution speed and performance^29^, its use for survival analysis remains largely unexplored). These are described as follows.

##### Random survival forests

 Random survival forests (RSFs) are based on the original random forest method^30^. Actually, their implementation follows the same principles. The base trees are grown (usually quite deeply) using bootstrapped data. The tree nodes are split using random feature selection. Lastly, the random forest output is calculated as the average of the individual tree predictors.

RSFs extend this concept by incorporating censoring information into the splitting rules when growing the trees. In other words, RSFs aim to split the tree nodes into branches with dissimilar survival^17,31^. Several splitting criterion have been proposed (e.g., conservation-of-events, log-rank score, log-rank approximation)^32^. However, the most widespread used rule (and therefore the one we chose) is the log-rank splitting rule. In this case, the rule splits the node by maximizing the log-rank test statistic. The larger the value, the greater the different between the two groups (i.e., branches) and the better the split is.

##### Survival support vector machines

 The aim of the original support vector machines (SVMs) is to find the hyperplane in the feature space that maximizes the margin between classes (i.e., maximum-margin hyperplane). They do so by mapping and transforming the instance space. The data instances that are closest to this hyperplane (i.e., the ones with the minimum distance to it) are the so-called support vectors^33^.

SVMs have been extended into RankSVMs, which are able to handle right-censored survival data. They cast survival analysis as a classification problem with an ordinal target variable. Instead of estimating survival times, they aim to predict risk ranks between patients^34–36^. Unfortunately, the objective function of ranking-based techniques depends quadratically on the number of instances, making them unfeasible for larger datasets. Survival SVMs (SSVMs) improve on them by efficiently modeling through the use of kernel functions^16,28^, allowing analyzing datasets of much larger size.

##### Extreme gradient boosting

 Gradient boosting machines (GBMs) are frameworks where the learning task is posed as a numerical optimization problem. Their objective is to minimize a loss function by adding weak learners (i.e., a learner whose performance is at least slightly better than random chance). The type of loss function depends on the problem. However, it must be differentiable, since it is optimized using a gradient-descent-like approach.

Extreme Gradient Boosting (XGBoost or XGB for short) is an optimized implementation of a GBM^37^. It uses decision (regression) trees as weak learners. In order to perform the gradient descent procedure, it calculates the loss and adds a tree to the model (always one at a time) that reduces it (i.e., follows the gradient). This is done by parameterizing the tree and modifying these parameters to move in the right direction by reducing the loss. The existing trees in the model are not changed. Trees are added until a fixed number is reached, until the loss reaches an acceptable level, or until no more improvement is achieved. XGB’s final output is given by the (weighted) sum of all the predictions made by all the individual trees.

In order to control over-fitting, XGB uses different regularization methods that penalize different parts of the algorithm, such as constraining the trees (e.g., number of trees, tree depth), weighting the updates (i.e., applying a learning rate), and using subsets of the data for generating each tree^29^. XGB is extremely popular in the ML community due to its high computational efficiency and great performance.

In all cases, the models’ parameters were optimized using randomized search of 25 different parameter settings. These were tested using a 10-fold cross validation targeted to maximize the $c$-index. Table 2 shows the parameter space explored for each model, aw well as the final parameter combination for each of them used for the rest of the analyses. For the CPH, RSFs, and SSVMs algorithms, we used the Python implementation available in scikit-survival (v. 0.12)^38^, while for XGB we used the Python implementation provided by Chen and Guestrin (v. 1.0.2)^37^.

**Table 2:** Models’ parameters.

Model	Parameter	Parameter space	Chosen value
RSFs	No. trees	{25, 50, 75, 100, 250, 500, 750, 1000, 1500}	500
	Max. depth	{1, 2, 3, 4, 5, 10, 15, 25, 50, 100, 250, 500}	250
	Min. samples split	{5, 10, 15, 20, 25, 50}	15
	Min. samples leaf	{1, 2, 3, 4, 5, 10, 25}	1
SSVMs	Kernel	linear, rbf, sigmoid	rbf
	$\alpha$	Logarithmic space ranging from 0.00001 to 10	0.113
	$\gamma \,^*$	Logarithmic space ranging from 0.001 to 1	0.717
	Booster	gbtree	gbtree
XGB	No. trees	{25, 50, 75, 100, 250, 500, 750, 1000, 1500}	50
	Max. depth	{1, 2, 3, 4, 5, 10, 15, 25, 50, 100, 250, 500}	5
	Learning rate	Logarithmic space ranging from 0.01 to 1	0.05
	Subsamples	{0.2, 0.3, 0.4, 0.5, 0.6, 0.7, 0.8}	0.6

Each model was parametrized using a randomized search of 25 different parameter settings with a 10-fold cross validation to maximize the $c$-index.* $\gamma$ is only relevant for the rbf kernel. It was ignored for the rest.

### Evaluation

These survival models output risk scores: a higher risk score means that there is a higher chance of the event of interest occurring early (in this case, death). These scores encompass the models’ target variable (time since diagnosis) together with its corresponding censorship indicator. However, they are given in an arbitrary scale. This means that the models are able to predict the sequence of events (i.e., which patients are more likely to pass away). Therefore, we evaluated the models using a metric suitable for such predicted risk scores while at the same time having a simple yet clinically meaningful interpretation: Harrell’s concordance index ($c$-index or *c* for short).

#### Concordance index ($c$-index)

The $c$-index is a measure of rank correlation between the models’ predicted risk scores and the observed time points (in the test data)^39–41^. It can be thought as a generalization of Kendall’s correlation $\tau$ tailored specifically for right-censored survival data^42,43^. In other words, the $c$-index quantifies how well a model predicts the ordering of patients’ death times. Furthermore, it is easy to interpret: the $c$-index estimates the probability that for a random pair of patients, the one having the lower risk score is the one who survives longer. A value of $c=0.5$ corresponds to the average performance of a random model (i.e., no predictive discrimination), while $c=1$ corresponds to a model capable of perfectly separating patients with different outcomes^39,40^.

Using 10-fold cross validation, we calculated the mean $c$-index (and corresponding 95% confidence intervals) of each model. Lastly, we compared the obtained $c$-index values using a paired Student t-test^44^ with Bonferroni correction for multiple comparisons.

### Explainability

Finally, we were interested in understanding how the models yielded their predictions. From the wide variety of explainability techniques available^45^, we decided to use SHapley Additive exPlanations (SHAP) values as proposed by Lundberg and Lee^46^, since they present several characteristics advantageous to our study. First and most importantly, SHAP values are model-agnostic. This means that they are not bound to any particular type of model (such as tree-based feature importance^47^), which was crucial to our analysis. Moreover, SHAP values present properties of local accuracy, missingness, and consistency, which are not found simultaneously in other methods^46,48^. Lastly, their implementation is easy to use, properly documented, and actively supported by an open-source community.

In order to understand how SHAP values work, we first need to explain the concept of Shapley value. Originally, Shapley proposed a game theory method for assigning fair payouts to players depending on their contribution to the total gain^49^. In a model prediction task, this translates to assigning a (quantitative) importance value to features depending on their contribution to a prediction. Thus, in our context, a Shapley value is defined as the average marginal contribution of a feature value across all possible feature coalitions. Under this definition, a Shapley value for a given feature value can be interpreted as the difference between the actual prediction and the average prediction for the whole dataset. It is worth noting that a Shapley value is *not* the difference of the predicted valued after removing its corresponding feature^19^.

The SHAP method computes the Shapley values and represents them as a linear model of feature coalitions^46,50^. Moreover, SHAP values make use of game theory’s Shapley interaction index, which allows to allocate payouts (i.e., importance) not just to individual players (i.e., features), but also among all pairs of them. This way, SHAP values are able to explain the modelling of local interaction effects, which could go unnoticed otherwise. This property is particularly important, since it allows the possibility of providing new insights into the model’s variables and the relations between them^51^.

Given the low variability of the evaluation metrics when using different folds of the data, we chose a random test data partition to compute the SHAP values of the CPH (as a reference) and of the best performing ML-based model using the Python implementation provided by Lundberg and Lee (v. 0.35)^46^. We used the SHAP values to obtain a visualization of the overall feature importance for the models. Then, we generated SHAP dependence plots for each model and compared how the features contributed to the corresponding models’ output. Lastly, we analyzed the most important interaction effects across features and their corresponding insights.

## Results

Table 3 shows the results of the traditional CPH analysis. On the one hand, according to this model and based on the *z* and *p* values of each feature, *age*, *pts*, and *ptmm*, were the three most important covariates. On the other hand, based on the obtained *p*-values, the features *rly* and *mor* had no significant impact on overall survival.

**Table 3:** Results of the CPH model.

Feature	HR	95% CI		*z*	*p*
*age*	1.055	1.054	1.057	80.191	0.000
*ratly*	1.604	1.434	1.794	8.269	0.000
*rly*	0.999	0.996	1.002	-0.672	0.501
*ptmm*	1.008	1.007	1.010	13.604	0.000
*ply*	1.021	1.012	1.031	4.456	0.000
***pts***					
I	1.000	–	–	–	–
IIA	1.096	1.045	1.149	3.801	0.000
IIB	1.352	1.265	1.446	8.804	0.000
IIIA	1.579	1.448	1.722	10.344	0.000
IIIB	2.250	2.034	2.490	15.707	0.000
IIIC	1.862	1.614	2.148	8.526	0.000
***grd***					
1	1.000	–	–	–	–
2	1.132	1.081	1.187	5.213	0.000
3	1.368	1.296	1.443	11.472	0.000
***mor***					
Ductal	1.000	–	–	–	–
Lobular	0.956	0.908	1.007	1.680	0.093
Mixed	1.043	0.961	1.133	1.843	0.313
Other	0.927	0.854	1.006	-0.655	0.071
***rec***					
Triple+	1.000	–	–	–	–
HR+	1.006	0.930	1.089	0.150	0.881
HR–	1.168	1.055	1.292	3.005	0.003
Triple–	1.473	1.348	1.609	8.577	0.000

*HR* hazard ration, *CI* confidence interval, *HR +* hormone receptor positive, *HR -* hormone receptor negative.

**Figure 1:**
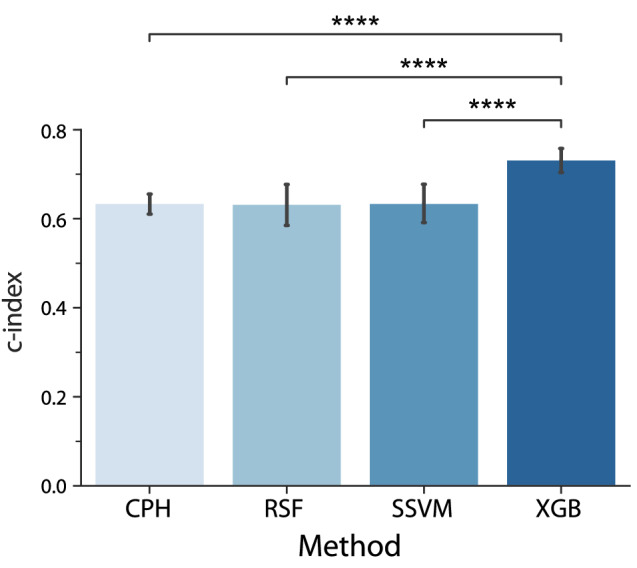
Average $$c$$-index of the different models using 10-fold cross-validation. Error bars represent 95% confidence intervals across folds. $$****p<0.0001$$. All other comparisons were non-significant ($$p>0.05$$) and are not shown for the sake of clarity.

Figure 1 depicts the bar plot of the quantitative evaluation of the models’ ranked survival predictions using the $c$-index. It shows its mean and 95% confidence intervals deviation obtained using 10-fold cross-validation for the different models. We can see that CPH, RSFs, and SSVMs yielded comparable values of $\sim 0.63$ to 0.64. However, XGB outperformed these methods with a *c* value of 0.73. This was confirmed by the paired Student t-test^44^ (with Bonferroni correction), which showed that the *c* value of XGB was significantly higher than the rest of the methods. All the comparisons between the other models were not significant.

**Figure 2:**
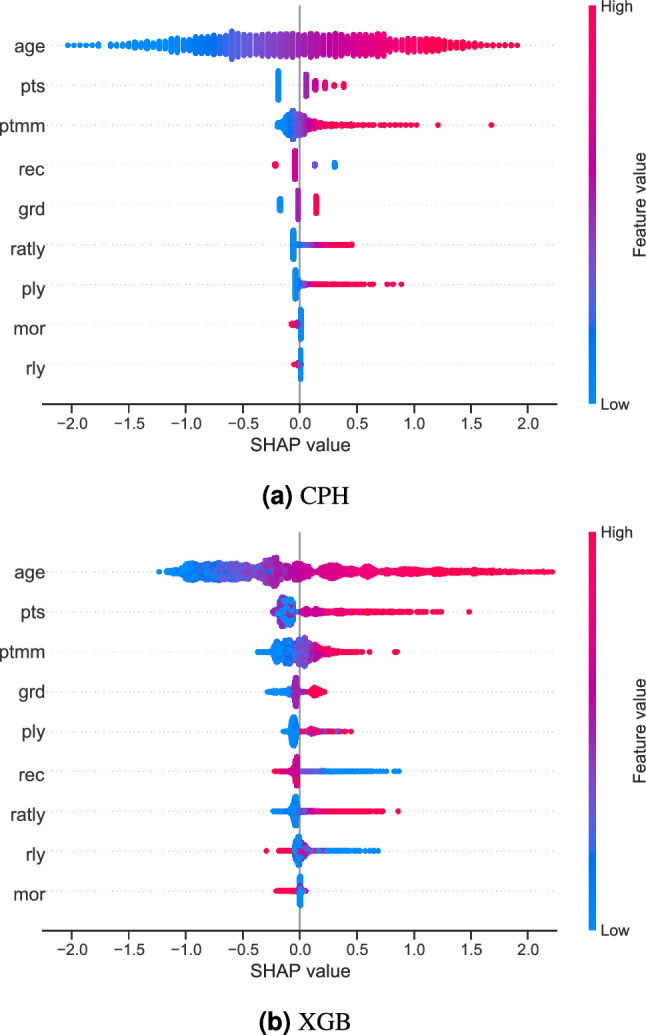
Summary plots for SHAP values. For each feature, one point corresponds to a single patient. A point’s position along the *x* axis (i.e., the actual SHAP value) represents the impact that feature had on the model’s output for that specific patient. Mathematically, this corresponds to the (logarithm of the) mortality risk relative across patients (i.e., a patient with a higher SHAP value has a higher mortality risk relative to a patient with a lower SHAP value). Features are arranged along the *y* axis based on their importance, which is given by the mean of their absolute Shapley values. The higher the feature is positioned in the plot, the more important it is for the model.

We computed the SHAP values of the CPH model (as a reference) and of XGB (the best performing ML-based model) for a given random partition of the data. Figure 2 shows their corresponding summary plots. Each point corresponds to an instance of the dataset (i.e., a single patient). Their position along the *x* axis (i.e., the actual SHAP value) represents the impact that feature had on the model’s output for that specific patient. Mathematically, for the task at hand (survival ranked predictions), this corresponds to the (logarithm of the) mortality risk relative across patients. In other words, a patient with a higher SHAP value has a higher mortality risk relative to a patient with a lower SHAP value. Moreover, features are arranged along the *y* axis based on their importance, which is given by the mean of their absolute Shapley values (higher position means higher importance). The color represents the features’ value.

**Figure 3:**
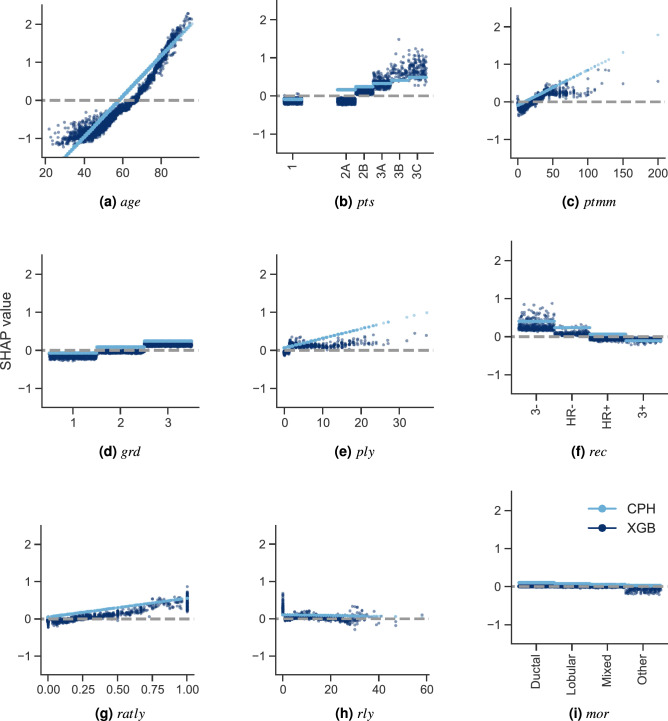
SHAP feature dependence plots. In the case of categorical variables, artificial jitter was added along the *x* axis to better show the density of the points. The scale of the *y* axis is the same for all plots in order to give a proper feeling of the magnitudes of the SHAP values for each feature (and therefore of their impact on the models’ output). In the case of the XGB model, the dispersion for each possible feature value along the *y* axis is due to interaction effects (which the CPH model is unable to capture).

Figure 3 compares the dependence plots of both models for all 9 features. These plots show the effect that a single feature has on the models predictions. Similarly to the previous plots, each point corresponds to an individual patient. In this case, a point’s position in the *x* axis corresponds to the value of the feature at hand. It is worth mentioning that in the case of categorical variables, artificial jitter was added along the *x* axis to each possible value to better show the density of the data points. A point’s position in the *y* axis corresponds to the SHAP value for that feature (i.e., how much does a value affect the model output for the prediction of that instance). In this case, the scale of all plots is the same to give a proper feeling of SHAP values magnitudes of each feature. On one hand, the data points in light color correspond to the CPH case. Notice how this approach is only able to model a linear effect between the features and their corresponding SHAP values. On the other hand, the dark color corresponds to the XGB model. In this case, we can see nonlinear behaviours, as well as the presence of interaction effects, which are represented by the vertical dispersion of SHAP values for a single feature value.

**Figure 4:**
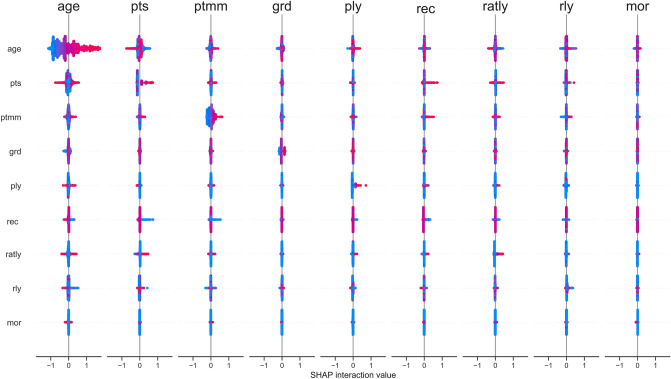
SHAP interaction values. The main effect of each feature is shown in the diagonal, while interaction effects are shown off-diagonal.

Speaking of interaction effects, we looked at them more in detail as modelled by the XGB approach and represented by the SHAP interaction values. Figure 4 shows the pairwise SHAP interaction values across all features. Here, the features’ main effects are seen in the main diagonal, while the interaction effects with other features are shown off-diagonal. It is worth mentioning that said effects are actually captured by the tails of the distributions (since at the center they have a SHAP value of zero). Notice that the overall model’s output is given by the sum of the whole interaction matrix. Therefore, the actual interaction effects (i.e., off-diagonal values) are divided in half (since there are two of each).

**Figure 5:**
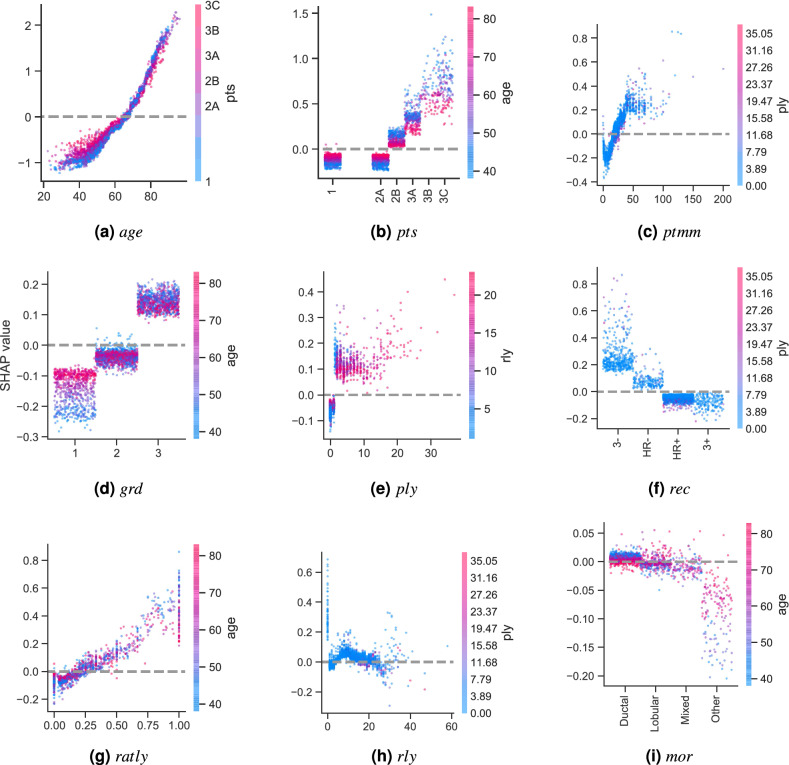
SHAP feature dependence plots of the XGB model showing the largest interaction effect for each feature. In the case of categorical variables, artificial jitter was added along the *x* axis to better show the density of the points. In this case, the scale of the *y* axis is *not* the same for all plots in order to better appreciate the interaction effects.

For each feature, we selected the case where the interaction effect with another one was the largest and plotted it as a SHAP dependence plot in Fig. 5. Here, the color represents the value of the interacting feature. We can see that *age* has the largest interaction effects in four cases (*pts*, *grd*, *ratly*, and *mor*), reaffirming the importance of its contribution to the model. It is followed by *ply* in three others cases (*ptmm*, *rec*, and *rly*). However, please note that in this figure, the scale of the *y* axis is *not* the same for all plots. This was set to better appreciate the interaction effects of all features, even of those that are relatively unimportant to the global model (such as those between *mor* and *age*).

## Discussion

In this study, we used a conventional multiple CPH regression and three different ML-based methods (RSFs, SSVMs, and XGB) to predict ranked survival in a relatively large population of 36,658 non-metastatic breast cancer patients. We compared the models’ performance using the $c$-index. We also computed SHAP values to explain the predictions of the reference model (CPH) and of the best performing ML model (XGB with a $c$-index$\sim 0.73$).

The first objective of this study was to compare the performance of the conventional CPH with that of different ML-based methods. The $c$-index of the CPH, RSFs, and SSVMs models yielded a value of $\sim 0.63$. The XGB model had a significantly higher performance with a $c$-index of 0.73 (Fig. 1).

These results show that in our case, ML-based models can perform at least as good (and in the case of XGB, even better) as the classical CPH approach on survival prediction tasks. They support similar findings from other oncological studies in literature. For example, Kim et al.^11^ compared the performance of CPH against RSFs and deep learning-based survival model in a set of 255 oral cancer patients, yielding $c$-index values of 0.69, 0.76, and 0.78, respectively. Datema et al.^14^ compared the performance of a CPH regression against RSFs using four different split criterion in a set of 1371 patients diagnosed with head and neck cancer between 1981 and 1998. They obtained a $c$-index value of 0.71 for the CPH regression and between 0.69 and 0.71 for the different RSF approaches. In the more specific case of breast cancer, Nicolo et al.^15^ tried to predict metastatic relapse after surgical intervention in 642 patients with early stage breast cancer. Their CPH and RSFs models yielded $c$-index values of 0.62–0.67 and 0.66–0.69, respectively. Moreover, they proposed a mechanistic model based on processes of metastatic progression, which yielded $c$-index values of 0.60–0.71. Omurlu et al.^13^ compared the performance of CPH against different variations of RSFs in a set of 279 breast cancer patients. Although they found that the approximate log-rank-based RSF approach yielded slightly higher $c$-index values (of 0.71), they concluded that the performance of all methods was very similar. Nevertheless, the performance of these novel ML-based methods (such as XGB) can (and should) be further confirmed with more studies that use large number of records, that are focused on different patient subpopulations and/or different types of cancer.

It could be alleged that the performance of the CPH regression model could be improved by extending it^52–54^. For example, nonlinear effects could be distinguished using *ad hoc* approaches (e.g., stepwise regression) and modelled using covariate transformations or specialized functions. Interactions between the model’s variables could be identified in an exhaustive manner (e.g., by examining all two-/three-way interactions) or based on subjective expertise to narrow the search^11,17^. Unfortunately, these alternatives have drawbacks of their own. Firstly, they are very resource-consuming tasks. More importantly, they are very likely to be overfitted to the task at hand, reducing the model’s generalizability and making it harder to compare against other previously published models.

The second objective was to provide better insights into why the models performed the way they did. Figure 2 shows the differences between the conventional CPH (which can be seen as a reference) and the best performing ML model (i.e., XGB) using SHAP values. The first thing to note is that the feature importance of both models is very similar. On the one hand, features *age*, *pts*, and *ptmm* were the most important for the models in general. On the other hand, features *mor* and *rly* were ranked at the bottom (although in a different order). The rest of the features were somewhere in between. Interestingly, this SHAP-value-based feature importance is consistent with the (implicit) feature importance given by the *p*-values of the traditional CPH analysis (Table 3).

SHAP values represent (the logarithm of) the relative risk of mortality: the higher the SHAP value, the more it contributes to predicted patient mortality. Figure 2 shows that in both CPH and XGB models, features tend to have long right tails. This suggests that there are more possible feature values that contribute to large risk scores (and therefore, higher chances of death), which intuitively makes sense.

SHAP feature dependence plots (Fig. 3) provide insights into global patterns of the model, as well as into single-patient variability. These plots reveal a few interesting observations worth discussing. In the first place, we can see why features *age*, *pts*, and *ptmm* were the most important ones: they yielded SHAP values with a large range, which dominated the models’ behavior. Secondly, we can also see why features *mor* and *rly* were the least important for the model: changes on their values had very little impact on their corresponding SHAP values, which were actually very close to zero.

Interestingly, SHAP feature dependence plots can also help us identify important turning points for the different features. For example, Fig. 3a shows that the CPH model considers $\sim 58\,\hbox {years}$ old as the turning point when age pushes its predictions from favoring a low to a high chance of mortality. In the case of the XGB model, this turning point is found at $\sim \,65$. For *ptmm* (Fig. 3c), the CPH model considers that the mortality risk increases indefinitely as a function of the tumor size. However, for the XGB model this increase is relevant until $\sim 45\,\hbox {mm}$. After that, it reaches a plateau^55–57^. This demonstrates the model’s capability of capturing nonlinearities present in the data. In the case of *ply* (Fig. 3e), the CPH model also assumes that the number of positive lymph nodes contribute proportionally to a higher mortality risk (including the case when the number of positive lymph nodes is zero). The XGB model shows something different. When there are no positive lymph nodes, *ply* contributes to a lower risk score, which aligns with clinical intuition and staging. It is only when the number of positive lymph nodes is $\ge 1$ that *ply* starts contributing to a higher risk score. The fact that SHAP values also allow us to investigate the impact of specific features on the model predictions is be very valuable, since it has been shown that this can be a complicated task even for experts due to high feature heterogeneity^58^.

Going more into the details of each approach, on the one hand Fig. 3 clearly shows the assumptions that the CPH model makes: it presumes that the features are completely independent and models their contributions linearly. This is reflected on the CPH model’s straight lines, where each feature value yields a single SHAP value. On the other hand, the XGB model is capable of capturing interaction effects between the features, which are shown as vertical dispersion for each feature value.

The (pairwise) interaction effects captured by the XGB model are shown exhaustively in Fig. 4. There are a few interesting instances worth discussing. The clearest (and largest) interaction effect occurs between the variables *age* and *pts*. Figure 5a,b show that patients that are between 20 and $\sim \,60$ years old with low *pts* (I and IIA) have a lower mortality risk than patients of the same age range with higher *pts* (IIB, IIIA, IIIB, and IIIC), which clinically makes sense. However, this difference is reduced considerably when patients are older than 60 years old. In the case of *grd*, Fig. 5d shows that for patients with *grd* 1, younger patients have lower mortality risk. This effect is lost in patients with *grd* 2 and 3. Figure 5e shows that when the number of *ply* is low (0 or 1), having a large number of *rly* ($\ge 5$) slightly increases the patient’s mortality risk. Lastly, in the case of *mor*, Fig. 5i shows that for ductal cancer, younger patients have a higher mortality risk. The effect of age on lobular and mixed tumors is unclear, but for all other morphologies, younger patients have a lower mortality risk. However, it should be noted that the effect of *mor* overall is quite small (actually, its importance was ranked the lowest). It is worth emphasizing that SHAP values allow elucidating the interaction effects modelled by the XGB model in a simple and straightforward manner, without needing to select arbitrary thresholds or any a priori knowledge of the data^51^. Considering the increasing complexity and volume of oncological data, it is crucial to have predictive models capable of capturing and providing an intuitive interpretation of interaction effects out-of-the-box with little effort of the researcher. This could be very valuable for clinical practice, since these models could, for example, provide crucial insights for identifying risk factors in patients or for evaluating new treatments.

As mentioned earlier, SHAP values present several advantages. First of all, they are model-agnostic, which means that they are not attached to any specific model and thus make a clear distinction between a model’s predictions and the explanations they provide^59^. This allows them to be used in combination with any ML approach (which was indispensable for our intent of comparing different models of a different type). Additionally, keeping the model separate can provide different degrees of interpretability and completeness for each (type of) explanation. Moreover, if a model needs to be updated or changed in a ML pipeline (which is not uncommon), it requires very little to no effort to adapt the explainability component, since the way in which the explanations are presented is kept the same^60^. SHAP values also present three key desirable properties not found simultaneously in other methods^46,48^: (1) local accuracy (correctly capturing the difference between the expected model output and the output of a given instance), (2) missingness (a missing feature receives an attribution value of zero), and (3) consistency (if changing a model increases the contribution of a feature value, its corresponding SHAP value should not decrease).

These advantages made SHAP an ideal choice for the purpose of this study. However, SHAP values should not be treated as a silver bullet. Although their implementation has been optimized for a few model architectures^50,61,62^ (which was beneficial for the XGB model), computing SHAP values for “generic” models can still be a very slow process (as was the case for the CPH model). This is especially important when the number of patients is very large and can limit their use in real-life use cases. Lastly, there are other emerging explainability techniques that could potentially be a more suitable choice depending on the application at hand^45,63–65^.

## Outlook and conclusion

In this study, we compared the performance of a conventional multiple CPH regression against three different ML methods (RSFs, SSVMs, and XGB) in a ranked survival prediction task using a dataset consisting of 36,658 Dutch non-metastatic breast cancer patients. Furthermore, we used SHAP values to open the models’ black-box and explain the difference in performance between a reference model (CPH) and the best performing ML model (XGB).

Our results showed that in the data at hand, ML-based approaches are capable of performing as good as a conventional CPH model or, in the case of the XGB model, even better. However, this comes at the cost of an increase in complexity/opacity. ML explainability techniques have arised as a solution for this issue. They can help us generate an explicit knowledge representation of how the model makes its predictions. In our case, SHAP values showed that the key difference between CPH’s and XGB’s performance can be attributed, at least partially, to the latter’s ability to capture data nonlinearities and interactions between features, which can have important contributions to the outputs. Moreover, it does so automatically and without any additional effort required by the researcher. Furthermore, SHAP values also allowed us to investigate the impact of specific features on the model predictions, which can be a complex task even for experts. This type of modelling frameworks could speed up the process of generating and testing new hypothesis on new (NCR) data, which could contribute to a rapid learning health system.

There is a growing body of literature that shows how cancer patients, clinicians, epidemiologists, and researchers in general can benefit from ML techniques. However, in order to bring these solutions closer to the clinic, users need to be able to trust these novel approaches. We believe that ML explainability techniques, especially those with a solid theoretical background behind them (like SHAP values), are key to bridging the gap between everyday clinical practice and ML-based algorithms.
